# Supplementary material for: An Experimental Hut Evaluation of PBO-Based and Pyrethroid-Only Nets against the Malaria Vector Anopheles funestus Reveals a Loss of Bed Nets Efficacy Associated with GSTe2 Metabolic Resistance
Source: Genes (Basel). 2020 Jan 29;11(2):143. doi: 10.3390/genes11020143 (PMC7073577; doi:10.3390/genes11020143)
Supplement: Supplementary file 1 [file genes-11-00143-s001.pdf]

genes

MDPI

Table S1. Linear mixed effect model analysis showing different sources of variations that may influenced the exophily.

	Value	Std.Error	p-Value
(Intercept)	16.249764	7.725620	0.0364
sleeper2	-4.932549	5.953246	0.4081
sleeper3	-0.300115	6.150593	0.9611
sleeper4	7.972452	5.568319	0.1534
sleeper5	4.356004	5.718595	0.4469
sleeper6	2.547911	5.839805	0.6630
Hut2	2.962492	5.533332	0.5928
Hut3	-5.224913	5.754090	0.3647
Hut4	4.504248	5.614402	0.4231
Hut5	-6.310800	5.844721	0.2813
Week2	19.753810	7.828069	0.0122
Week3	8.457334	7.436382	0.2565
Week4	-6.289393	8.023420	0.4338
Week5	4.201448	7.381160	0.5697
Week6	-5.579260	7.661576	0.4671
Week7	-3.276825	8.130004	0.6872
Week8	9.517598	7.753166	0.2207
Week9	17.027261	8.521754	0.0468
Week10	-5.011175	7.652644	0.5132

Table S2. Linear mixed effect model analysis showing different sources of variations that may influenced the mortality.

	Value	Std.Error	p-Value
(Intercept)	13.432237	8.932373	0.1339
sleeper2	2.261362	6.402623	0.7242
sleeper3	-6.058720	6.611833	0.3603
sleeper4	2.695107	5.986638	0.6530
sleeper5	4.570812	6.153108	0.4582
Hut2	5.200011	5.953404	0.3832
Hut3	2.809049	6.189717	0.6503
Hut4	5.747833	6.040292	0.3422
Hut5	5.557012	6.289644	0.3778
Week2	8.142864	8.423385	0.3346
Week3	-6.702629	7.995142	0.4026
Week4	2.918904	8.626829	0.7354
Week5	5.451842	7.936171	0.4927
Week6	-4.389710	8.239292	0.5946
Week7	-8.807101	8.743382	0.3147
Week8	0.442853	8.344017	0.9577
Week9	4.040681	9.164440	0.6596
Week10	-8.851122	8.229572	0.2831

Table S3. Linear mixed model effect analysis showing different sources of variations that may influenced the Blood feeding.

	Value	Std.Error	p-value
(Intercept)	22.223943	9.227391	0.0167
Sleeper 2	3.777416	6.544700	0.5643
Sleeper 3	6.917576	6.758355	0.3070
Sleeper 4	4.361940	6.119352	0.4766
Sleeper 5	1.186007	6.289840	0.8506
Hut2	-4.436482	6.085664	0.4667
Hut3	-6.593280	6.327146	0.2984
Hut4	-13.011677	6.174456	0.0360
Hut5	-4.729087	6.429454	0.4627
Week2	5.612935	8.610579	0.5151
Week3	6.941759	8.172390	0.3964
Week4	1.370215	8.818114	0.8766
Week5	2.453404	8.112139	0.7626
Week6	-9.002432	8.422083	0.2861
Week7	-2.650813	8.937378	0.7670
Week8	7.169607	8.529541	0.4014
Week9	15.988995	9.367762	0.0891
Week10	11.157829	8.412140	0.1859

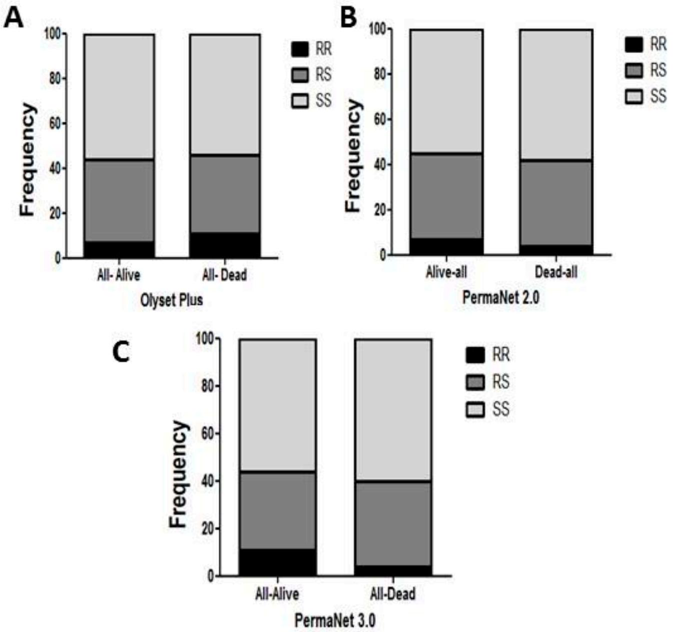

Figure S1. Impact of the *L119F-GSTe2* mediated metabolic resistance on bednet efficacy looking at the mortality rate after exposure to LLINs: (**A**) Genotype distribution of L119F-GSTe2 between alive and dead mosquitoes after exposure to Olyset Plus showing no association; (**B**) Genotype distribution of L119F-GSTe2 between alive and dead mosquitoes after exposure to PermaNet 2.0 showing no association; (**C**) Genotype distribution of L119F-GSTe2 between alive and dead mosquitoes after exposure to PermaNet 3.0 showing a trend of increased ability to survive in resistant mosquitoes but ($P > 0.05$). Only 5 dead mosquitoes were recorded for Olyset preventing assessment for this LLIN.

© 2020 by the authors. Licensee MDPI, Basel, Switzerland. This article is an open access article distributed under the terms and conditions of the Creative Commons Attribution (CC BY) license (http://creativecommons.org/licenses/by/4.0/).
